# The neuroimmune response during stress- a physiological perspective

**DOI:** 10.1016/j.immuni.2021.08.023

**Published:** 2021-09-14

**Authors:** Hedva Haykin, Asya Rolls

**Affiliations:** Department of immunology, Rappaport Faculty of Medicine, Technion - Israel Institute of Technology, Haifa, 3525422, Israel

## Abstract

Stress is an essential adaptive response that enables the organism to cope with challenges and restore homeostasis. Different stressors require distinctive corrective responses in which immune cells play a critical role. Hence, effects of stress on immunity should vary depending on the stressor. Indeed, epidemiologically, stress can induce either inflammation or immune suppression. However, in the absence of a conceptual framework, these effects appear chaotic, leading to confusion. Here, we examine how stressor diversity is imbedded in the neuroimmune axis. Stressors differ in the brain patterns they induce, diversifying the neuronal and endocrine mediators dispatched to the periphery generating a range of potential immune effects. Uncovering this complexity and diversity of immune responses to stressors will allow us to understand the involvement of stress in pathological conditions, identify ways to modulate it, and even harness the therapeutic potential embedded in an adaptive stress response.

## Introduction

Stress is the organism’s response to any change that causes physical or psychological strain and pressure. Stress can generally be considered as the experience of anticipating or encountering adversity, and stress responses are the body’s nonspecific reactions (e.g., physiological, behavioral, and emotional) to the experience of stress. Thus, stress is an expansive, all-embracing term encompassing any deviation from homeostasis. Hans Selye, the father of stress research, characterized stress as ‘a scientific concept, which has received the mixed blessing of being too well known and too little understood’ (Selye, 1936). This description also applies to the effects of stress on immunity; while such effects undoubtedly exist, their exact nature remains unclear. Stress has been associated with both marked inflammation and immune suppression (Segerstrom and Miller, 2004). Epidemiologically, stress is associated with the emergence and exacerbation of chronic inflammatory disorders, as seen in systemic lupus erythematosus, multiple sclerosis, rheumatoid arthritis and inflammatory bowel disease (Herrmann et al., 2000). In contrast, stress induced mediators such as the neurotransmitter, noradrenaline or glucocorticoids, common stress hormones, induce immune suppression and in fact, glucocorticoids are used as an immunosuppressant for the treatment of these same conditions (Rhen and Cidlowski, 2005). Such contradictory effects of stress on the immune system, have led to confusion in the field, limiting our capacity to harness the stress response for therapeutic interventions.

However, this apparent discrepancy can be resolved, at least in part, if we recognize that stress is not a uniform phenomenon. Stressors vary in their origin (physical or psychosocial), their duration (acute or chronic), and their intensity (mild, moderate or intense). Stress heterogeneity also stems from individual physiological and psychological differences and factors such as the organism’s inflammatory, metabolic and emotional state, which affect the individual’s perception of stress and its physiological outcome (Koolhaas et al., 2010). Therefore, attributing a uniform manifestation to stress limits our ability to gain a mechanistic understanding of the phenomenon.

The attempt to produce a uniform view of stress reflects an understanding of stress articulated by Selye, who defined stress as “the non-specific response of the body to any demand” (Selye, 1936). Selye used the term ‘non-specific’ to indicate that, regardless of the nature of any demand or stressor, all responses to stress share some common characteristics. The “General Adaptation Syndrome” proposed by Selye was a pioneer framework describing a uniform stress response characterized by three phases: a nonspecific mobilization phase, which promotes sympathetic nervous system activity; a resistance phase, during which the organism attempts to cope with the threat; and an exhaustion phase, which occurs if the organism fails to overcome the threat and depletes its physiological resources (Selye, 1936). While the mechanistic concept proposed by Selye of neuroendocrine control of the pituitary-adrenal humoral axis has been expanded and has been generally validated (Miller, 2018), Selye’s perception of the stress response as uniform response to challenge, has been disputed over the past decades (McEwen and Stellar, 1993; Fink, 2017). The perception of the stress response has shifted from the notion of restoring the system to its original setting, to the concept of allostasis, in which the stressful experience is incorporated to form a new homeostatic and dynamic set-point to generate a physiological equilibrium and development (McEwen and Stellar, 1993). Thus, stress is increasingly perceived as a required mechanism for growth and development rather than a mere threat to the organism’s homeostasis (Crum *et al*., 2020). New ideas and findings indicate that life experiences, genetics, and behavior affect the nature of the stress response, highlighting the ability of distinct stressors to exert markedly different physiological and psychological reactions that vary between individuals (Forkosh et al., 2019). This change is reminiscent of the transition that occurs in the field of cancer research, which has evolved to view cancer as a collection of pathologies, each characterized by its unique etiology and physiology. This conceptual shift has transformed the investigation of cancer, allowing the field to uncover specific physiological mechanisms and adapt therapeutic strategies. Similarly, by defining the diversity of the stress reactions, uncovering different mechanisms of stress-induced physiological mechanisms and their psychological and physical implications, we will be able to understand and modulate the detrimental effects of stress and utilize its therapeutic potential. Such a conceptual change will require, in part, adjusting the relevant terminology to describe specific types of stressors and to define specific axes on which stressors and stress responses are analyzed (Richter-Levin and Sandi, 2021).

**Figure 1:**
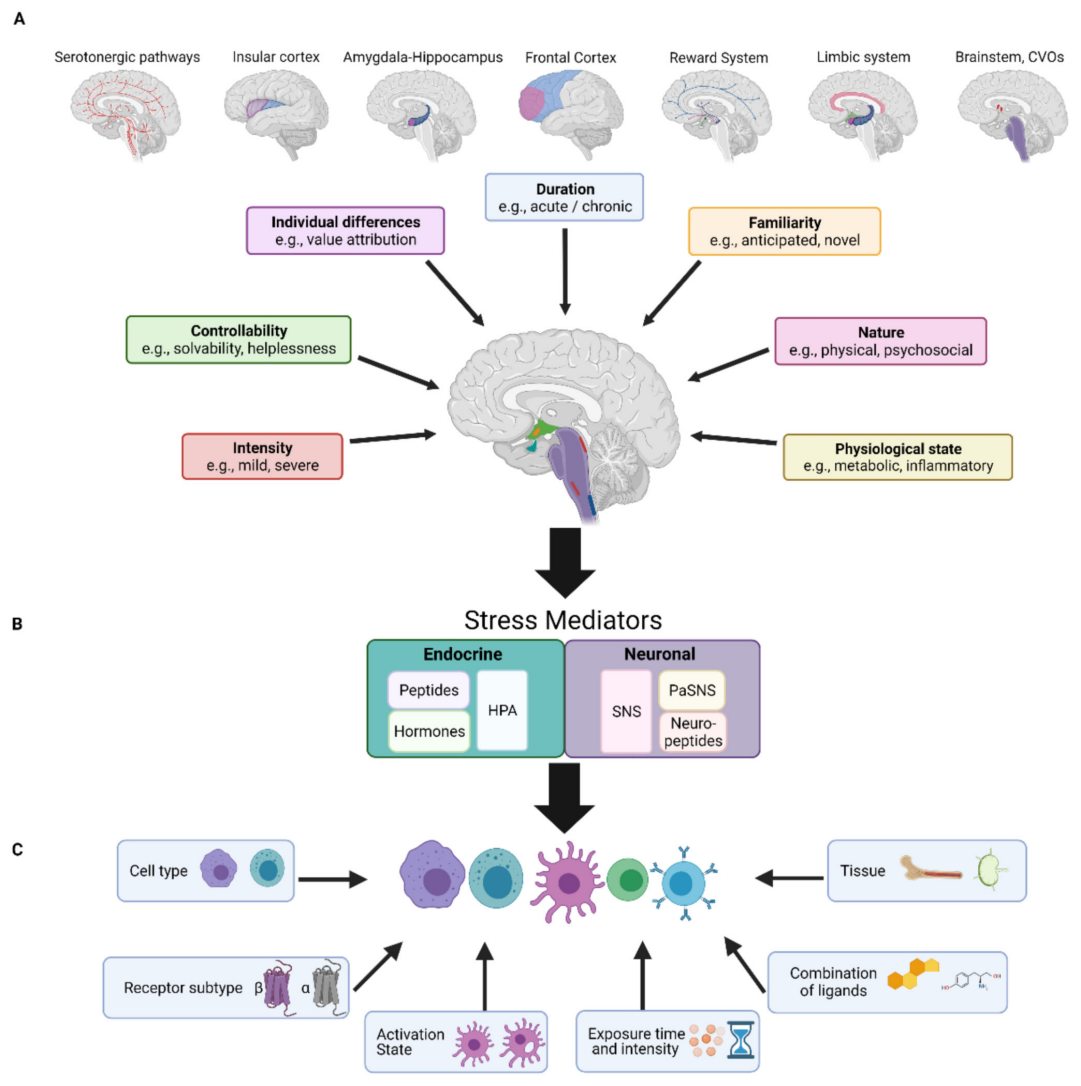
An overview of the variables influencing the effects of stress on the immune system. The stress response can be regulated at three levels by numerous variables, all of which ultimately alter the immune response and the immunophenotype produced. **A**. The characteristics of the stressful stimulus. Key stress characteristics are shown in boxes, along with their associated brain regions. Some brain regions are involved in various forms of stress, such as the reward system, which is attuned to predictability, duration, and controllability and the frontal cortex, which is receptive to predictability in addition to the controllability and duration, shown here. The brainstem, CVOs, and limbic system are sensitive to the nature of the stressful stimulus and to the physiological state of the organism. The overall activity of the brain effectively builds a specific neuromatrix for each stressor. The information reaching the brain is integrated at two main regions – the hypothalamic PVN nucleus and the brainstem nuclei (e.g., LC, NST, RVLM). These regions produce both endocrine and neuronal outputs. **B**. The neuronal or endocrine mediators. Two main stress-mediating systems, the endocrine (turquoise) and the neuronal (purple) responses, are represented in boxes. Listed within the boxes are the major components com rising each system, all of which participate in the mediation of stress, based on the input received from the brain. **C**. Characteristics of the responding immune populations. Factors that influence immune-cell responsiveness are noted in boxes. The combined effects of these factors will determine the resultant stress-induced immune response. *CVOs: circumventricular organs; HPA: hypothalamus- pituitary- adrenal axis; SNS: sympathetic nervous system; PaSNS: parasympathetic nervous system; LC: Locus coeruleus; NST: nucleus of the solitary tract; RVLM: rostral ventrolateral medulla*.

In this review, we will attempt to provide an overview of the different factors that can contribute to the diversity of stressors, the stress responses induced by the brain, and their potential effects on the immune system (Fig 1). We will examine the interactions between the brain and the immune system at three different levels: the initiator, the mediators, and the targets. The brain is the initiator of the stress response, capturing external and internal sensory inputs. The brain processes these inputs and integrates them to produce an orchestrated response designed to cope with the perceived challenge and anticipate the predicted deviation from homeostasis (Fig 1A). Mediators then deliver the information from the brain to the periphery in order to execute an orchestrated physiological response. These mediators comprise descending pathways, including the endocrine pathway, the hypothalamic–pituitary–adrenocortical (HPA) axis, which secretes glucocorticoids, in addition to the autonomic nervous system (ANS), specifically the sympathetic nervous system (SNS), which releases noradrenaline (NA) and neuropeptides (Fig 1B). Finally, within the context of this review, the target will be the immune system. The mechanisms activated by these mediator pathways are not binary as they have several levels of complexity manifested in the different secreted factors, their concentration and their combinations. These include, for example, the concentration of NA vs adrenaline or the various neuropeptides that can be secreted by peripheral neuron (Besedovsky and Rey, 2007). In addition to neuronal mediators and the HPA axis, the brain regulates other endocrine mediators that can control immune processes, such as prolactin and oxytocin (Wu et al., 2014; Carter et al., 2020). The opiate system, mainly associated with stressors accompanied by pain, can also affect immune activity (Moyano and Aguirre, 2019). Thus, the combined activity of the various mediators (e.g neurotransmitters, hormones, opioids, neuropeptides) generates a repertoire of potential signals that can each yield a different outcome depending on the target, the immune cell. Immune cells carry receptors for such neurotransmitters, neuropeptides and hormones (Besedovsky and Rey, 2007; Glaser and Kiecolt-Glaser, 2005). The composition of these receptors varies depending on cell type, developmental state, the tissue in which they are located, and the activation state of the cells, manifesting a unique profile for individual cells (Pavlov and Tracey, 2017) (Fig 1C. Thus, the functional and immunological outcome of a specific stress response depends on multiple levels of diversification embedded in brain, the mediators it secretes, and the target immune cells.

## The initiator-the brain

The identification and classification of a stressor is first determined by the brain. Upon detection of a stressor, the brain evaluates it in the context of the organism’s physiological and psychological state, and induces a corrective response. Since stressors vary in their nature, their context, and between individuals, the brain’s activity should also vary following exposure to different types of stress (Sousa, 2016). Stressors can be divided based on several factors, for example, type of stressor (physical, psychological), its intensity and duration of exposure (acute, chronic), the physiological state of the organism experiencing the stress, the individual’s predisposition (experience and genetic makeup), the value attributed to the stressful experience (positive, negative) and the degree to which the stress may be controlled and predicted. Thus, each stressor, whether it is generated by the brain (psychological) or detected by it (physical), is expected induce a distinct pattern of brain activity, which is reflected in the specific response that is initiated (Fig 1A).

Although stress clearly has widespread effects throughout the brain, there are several key brain regions that are the most relevant in this context. These include mainly the hypothalamus, which regulates the neuroendocrine pathways modulating hormone secretion by the pituitary gland and areas that regulate the sympathetic and parasympathetic (PaSNS) systems (Ulrich-Lai and Herman, 2009). The endocrine outputs, mainly the HPA axis, are regulated specifically by corticotrophin-releasing hormone (CRH) neurons in the hypothalamic paraventricular nucleus (PVN) (Miller, 2018). CRH activates adrenocorticotropin (ACTH) secretion from corticotropic cells in the anterior pituitary gland, which in turn stimulates corticosteroid release and production from the adrenal cortex (Ulrich-Lai and Herman, 2009). Other neurons in the PVN that control SNS activity project onto the autonomic relay nuclei of the brainstem, the rostral ventral lateral medulla (RVLM) (Ulrich-Lai and Herman, 2009), and the intermediolateral spinal columns (Zheng et al., 1995). Non-hypothalamic brain sites such as the noradrenergic Locus Coeruleus (LC) also affect the sympathetic outflow (Godoy et al., 2018). Indeed, brain areas such as the adrenergic cell group in the C1 region in the medulla oblongata, which act as a principal gateway for integrating autonomic responses, has been shown to regulate immune responses during stress and to activate an anti-inflammatory pathway (Abe et al., 2017). The activity of these areas that directly control the peripheral output is regulated by the inputs they receive from other brain areas so that the combined response of the entire brain activity can ultimately determine the endocrine and neuronal signals met by the immune cells. This brain activity can vary both between stressors and individuals. In this section, we provide specific examples of different parameters that can be applied to distinguish between stressors, their potential representation by the brain, and their functional outcomes.

Stressors can originate from different sources (physiological or psychological) and differ in the type of sensory input that mediate them (e.g., the visual image of a wild boar or an interoceptive pain signal). In the demonstrator-observer stress paradigm, a repeated electric foot shock is delivered to a group of mice (demonstrators). This is a commonly used physical stress model (Li et al., 2019). However, in some experiments, another group of mice (observers) witness the demonstrator mice as they receive the foot shock. The observer mice also experience stress, but a psychological one. Observers develop some stress-related physiological responses and freezing behavior similar to those mice that received the physical shock (Jeon et al., 2010). However, the brain activity associated with the stress response in the observer mice is different. For example, activation of the anterior cingulate cortex (ACC) is required to induce the stress response specifically in the observer mice (Jeon et al., 2010). The ACC mediates connections between the “emotional” limbic system and the “analytical” prefrontal cortex (Mohanty et al., 2007; Stevens et al., 2011). This network of interactions between the ACC and medial prefrontal cortex (mPFC) can regulate autonomic and neuroendocrine effector pathways, and thus, modulate inflammatory states (Ulrich-Lai and Herman, 2009). As a side note, since the interactions between the brain and the immune system are bidirectional, it was shown that activity within this neuronal network is affected by systemic inflammation, specifically by interleukin (IL)-6 (Marsland et al., 2017; Ho et al., 2021).

The nature of the strains posed on the organism by psychological and physical stress are distinct and thus expected to recruit different physiological and immunological mechanisms to cope with the challenge. Indeed, studies that evaluate the effects of psychological vs physical stress on the immune response, demonstrate that these stressors differ in their effects on plasma concentrations of IL-1β and IL-4 (Du Preez et al., 2020). Moreover, it has been hypothesized that while physical stress will require immune cellular mobilization to peripheral tissues, psychological stress may mainly require adaptation of the brain’s immune compartment, since immune cells are required for effective brain activity and specifically the ability to cope with psychological stress (Schwartz and Baruch, 2012). For example, in the model of unpredictable chronic mild stress, modulation of microglial (the resident myeloid population of the brain) activity in the hippocampus affects local neurogenesis (Du Preez et al., 2021), which in turn, can impact the long term outcomes and development of depression (Kreisel et al., 2014). It has been shown in mice that stress-induced lymphocyte trafficking to the brain compartment, improves behavioral responses of the mice and restores hippocampal brain-derived neurotrophic factor levels (Lewitus et al., 2008). Exposing T cell-deficient mice to predator odor results in their maladaptive behavioral response (e.g startle response and avoidance behavior) to the stressor compared to their wild-type counterparts. This maladaptive response could be reversed by introduction of T cells reactive to central nervous system (CNS)-associated self-proteins (Cohen et al., 2006). Thus, physical and psychological stressor differ in the type of sensory inputs required for their perception, the neuronal networks they activate, and the extent to which they affect the brain’s immune compartment relative to peripheral changes in immunity.

Familiarity with the stressor has significant implications on the nature of the stress reaction. A familiar stimulus allows the subject to anticipate potential outcomes and prepare for them. Thus, a familiar stressor will activate areas related to memory such as the hippocampus, amygdala and cortical representations (Kim and Cho, 2020). These areas have been repeatedly associated with immune modulation. For example, neurons in the central nucleus of the amygdala (CeA) are connected to the splenic nerve; ablation or pharmacogenetic inhibition of these neurons reduces plasma cell formation, whereas pharmacogenetic activation of these neurons increases plasma cell abundance after immunization (Zhang et al., 2020b). In the context of a familiar stressor, which has been encountered before, and hence, expected to have a relevant B cell representation, brain-spleen neural connection that autonomically enhances humoral responses may enable the organism to mount an effective immune memory response.

Another brain area differentially modulated by familiar and novel stressors is the reward system. Familiar stressors enable the brain to generate predictions based on past experience to anticipate the potential impact of the stressor on the organism. The reward system is essential in generating such predictions and through its wide projections in the brain, can modulate the nature of the stress response. For example, levels of endocrine mediators such as serum norepinephrine and corticosterone, which are induced during chronic stress, can be attenuated by activation-specific components in the reward system (Xu et al., 2021). Optogenetic stimulation of dopaminergic neurons in the ventral tegmental area (VTA), which project to the mPFC, can rescue anxiety-like behavior induced by chronic stress (Xu *et al*., 2021). It can also modulate peripheral immune activity and the organism’s response to bacterial infections (Ben-Shaanan et al., 2016) and tumors (Ben-Shaanan et al., 2018; Xu *et al*., 2021). Thus, the brain’s anticipatory response can affect the nature of the physiological and immune reaction to stress.

The familiarity with the stressor is also related to one’s ability or perception of the ability to control a given stressor. The degree of control over a given situation is also represented in the brain (Lucas et al., 2014; Limbachia et al., 2021). It has been shown that activity of CRH neurons in the hypothalamus during experimental stress in mice, depends on their ability to control the outcome (Daviu et al., 2020). High outcome control increases anticipatory activity, whereas stressors with no outcome control prevents the emergence of such activity in the CRH neurons (Daviu *et al*., 2020). Controllable stress reduces its aversiveness, as evident by decreased stressor-related responses across threat-related regions, notably in the bed nucleus of the stria terminalis (BNTS) and anterior insular cortex (Limbachia *et al*., 2021). Moreover, stress induces activation of a serotonergic nucleus, the dorsal raphe nucleus (DRN); however, when a stressor is controllable, this activation is inhibited by the mPFC (Amat et al., 2005). Activation of the serotonergic DRN increases active coping with inescapable stress in rodents (Nishitani et al., 2019). Serotonergic activity affects the SNS and plays a role in thermoregulation (Hale et al., 2011) and can affect immune activity (Evans et al., 2015). Thus, the predictability and degree of control over a stressful situation, affect the processing of the stressful situation by the brain, and the associated anxiety, and alters the descending stress-induced mediators to the periphery.

The individual genetic and environmental makeup impacts the processing and perception of stressful the stimuli, resulting in divergent outcomes between subjects exposed to a similar stressor (Chikazoe et al., 2014; Crum *et al*., 2020). Response to stress appears to have strong innate encoding as shown in a study that differentiates between four identity domains (“personality types”) in mice based on their behavior in an enriched environment. These identity domains are stable over time and developmental stages and represent specific transcriptomic patterns in the brain, mainly in the amygdala, insular cortex and mPFC (Forkosh *et al*., 2019). Analysis of the immune response to stress of animals exhibiting the same identity domains may be especially valuable of classification of the immune outcomes of stress.

Individual differences in the connectivity between brain structures and gene expression profiles in the brain can alter the way a stressor is interpreted by the brain, and hence, the nature of the messages propagated to the periphery, including the immune system. For example, hippocampal connectivity with a network of other brain structures including the hypothalamus predicts stronger feelings of stress (Goldfarb et al., 2020) so that a similar stressor will induce different stress reactions. These brain networks control the peripheral neuro-endocrine mediators and thus, the peripheral immune response (Glaser and Kiecolt-Glaser, 2005; Schiller et al., 2021b). Some aspects of these individual predispositions appear to be genetic (Ebner and Singewald, 2017). However, others depend on past experience. Stress during critical or sensitive periods of early development has lasting effects throughout life altering the neuronal component of the brain, as well as the immune compartment in the brain and in the periphery (Danese and S, 2017). Early life exposure to stress has been shown to remodel the HPA axis, and in turn, alter immune responses to stress at adulthood (van Bodegom et al., 2017). Specifically, it has been demonstrated that glucocorticoid exposure in early life results in diminished CD8^+^ T cell responses in adulthood (Hong et al., 2020). Thus, the individual differences that arise from genetics and life experiences affect the sensory, processing and modulation of the stress response.

Analysis of the effects of stress on immunity should consider gender as a significant factor that affects the psychological and physiological aspects of stress (Babb et al., 2013; Hodes et al., 2015). However, the specific effects vary with the stressor. For example, acute restraint stress activates more corticotropin-releasing factor (CRF) neurons and BNTS in adult female rats as compared to males (Babb *et al*., 2013). Females are more sensitive to a chronic variable stress manipulation, and this increased sensitivity is linked to epigenetic regulation of the CRF signaling pathway in the nucleus accumbens, which is part of the reward system (Hodes *et al*., 2015). In contrast to the effects of stress in adulthood, maternal stress early in gestation increases stress responsiveness only in male offspring, an effect that is associated with epigenetic modifications of the CRF gene (Mueller and Bale, 2008; Thorsell and Natt, 2016). These stress specific differences are layered on top on the baseline differences in immune activity between the genders.

Although all the factors mentioned above will affect the stress response and its effects on the immune system, the most studied aspect of the effects of stress on immunity is the duration of the stress (Segerstrom and Miller, 2004). Acute stress induces a rapid response to restore homeostasis ; however, when exposure to the stressor is prolonged, it begins to involve other systems, and indeed, it has been shown to have distinct manifestations in the brain and distinct effects on physiological processes (Sousa, 2016). In terms of the immune effects, the duration of stress exposure determines the damage to the immune system and its reversibility (Sarjan and Yajurvedi, 2018). Generally, acute stress is considered to enhance the immune response, while chronic stress suppresses immune system function (Dhabhar and McEwen, 1997; Dhabhar, 2009). Yet, both acute and chronic stress can elicit proinflammatory immune responses and anti-inflammatory responses, as well (Abe *et al*., 2017; Miller et al., 2019). For example, repeated injections, a physical stress, or social isolation, a psychosocial stress, or any combination of those, leads to distinct corticosterone responsivity, and thereby results in different amounts of cytokines such as IL-2 and IL-4 (Du Preez *et al*., 2020). Prolonged stress results in a continuous low-grade inflammation, leading to wear and tear of tissue (Liu et al., 2017; Sarjan and Yajurvedi, 2018). Similarly, effects of stress on tissue permeability in the gut, can induce low grade inflammation by increasing the exposure to microbes, creating an intestinal barrier defect, and altering the immunological tolerance in this tissue (Karl et al., 2017). This has been shown to result in increased antibacterial IgA and irritable bowel syndrome (Gao et al., 2018). Indeed, multiple epidemiological studies have demonstrated low grade inflammation in people suffering from chronic stress (Segerstrom and Miller, 2004).

A series of studies has demonstrated that various socio-environmental risk factors, which reflect a chronic situation, including poverty and bereavement, are associated with specific gene expression patterns, characterized by upregulated transcripts involved in inflammation, and downregulated transcripts involved in antiviral responses (termed conserved transcriptional response to adversity; CTRA). In the face of stressful experiences, this transcriptional program is thought to promote chronic low-grade inflammation, and thus provides a mechanistic link between stress and the development of inflammation-related diseases (Dieckmann et al., 2020). An interesting emerging question is whether such low-grade inflammation has an adaptive role in the stress response.

In terms of brain activity, previous studies have characterized some differences between acute and chronic stress. With repeated exposure to stress, CRH neurons of the PVN display cellular, synaptic, and connectional plasticity, which have been suggested to maximize the ability of the HPA axis to maintain response vigor and flexibility (Ramot et al., 2017). Nevertheless, how these changes in brain activity ultimately affect the immune response, is still unclear.

In summary, the stress response is a product of the functional outcome from the collective brain activity reflecting different factors, including the nature, intensity, familiarity, predictability and duration of the stressor, as well as the organism’s physiological state and personal disposition. These factors determine the overall activity in the brain, manifested in the outflow of information to the periphery.

## The mediators - endocrine and neuronal pathways

**Figure 2:**
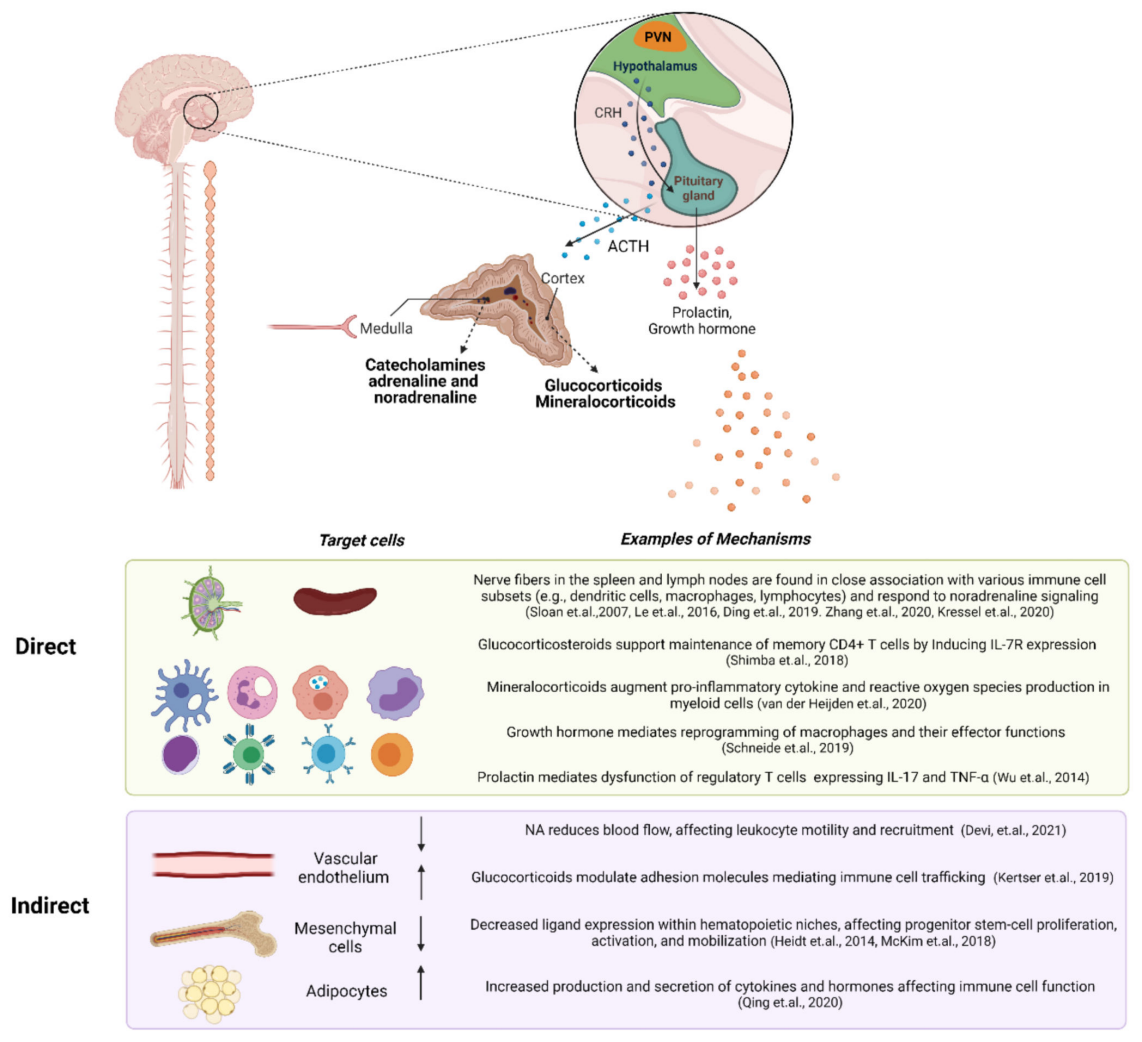
Examples of direct and indirect effects of stress on the immune system. Endocrine and neuronal signals can affect immune-system function, by acting either directly on immune cells or indirectly via the surrounding tissue. The autonomic nervous system innervates the adrenal medulla, inducing it to secrete catecholamines into the bloodstream. Within the lymph nodes and spleen, various immune subsets remain in close association with sympathetic fibers. Signals received by immune cells from nerve fibers in these compartments may affect immune-cell gene expression, maturation, migration, proliferation, differentiation, activation, and other functions. The indirect effects of stress on immune cells are mediated by the cells comprising the various organs. For example, stress modulates the expression of adhesion molecules on endothelial cells, thereby affecting immune cell trafficking. Stress also reduces blood flow and interrupts the locomotion of leukocytes into tissues via calcium signaling. Within hematopoietic niches, stromal cells affect immune cell maturation and mobilization through the expression of specific ligands. Upon exposure to stress, adipocytes secrete IL-6 which affects immune cell activity and recruitment. PVN: paraventricular nucleus; CRH: corticotrophin hormone; ACTH: adrenocorticotropin hormone.

Both endocrine and neuronal pathways secrete peripheral mediators that can regulate immune activity either by directly affecting immune cell activity or their surrounding tissue (Fig 2). Although both regulated by the brain, the HPA axis and the neuronal SNS pathways differ in their temporal and spatial resolution. While the neuronal signals can travel rapidly and reach very specific sites, the endocrine pathway depends on the dynamics of blood circulation to reach its targets, usually involving a secretion cascade, with one hormone inducing the secretion of the subsequent one.

The HPA axis has been extensively reviewed (Miller, 2018). Thus, we focus here on other hormones, beyond those of the classical stress response (ACTH and glucocorticoids), which are secreted to the periphery during stress. For example, the pituitary secretes alpha-melanocyte stimulating hormone (Lookingland et al., 1991), a neuropeptide known for its anti-inflammatory effects (Zhang et al., 2020a; Svea Kleiner, 2021). Arginine vasopressin (AVP), controls resorption of water in the renal ducts and can influence the regulation of ACTH secretion by the pituitary gland to impact the brain-induced stress response (Zelena et al., 2009; Gray et al., 2012). However, AVP also has some immunological properties and acts as a chemoattractant for monocytes (Wiedermann et al., 2018). The secretion of these and other hormones varies between stressors. Stressful conditions involving metabolic pressure are known to induce the secretion of growth hormone (Jezova et al., 2007), which is shown to affect immune cell activation (Schneider et.al., 2019). Even the secretion of the classical stress hormones such as ACTH and corticosterone depend on the context of the applied stress. For example, lactating females taking care of pups exhibit blunted neuroendocrine responses to many common stressors (Deschamps et al., 2003). However, when the stressor includes a perceived danger to the pups (for example, a predator odor) plasma ACTH and corticosterone responses are significantly increased, reaching levels comparable to that of virgin females (Deschamps *et al*., 2003). Hence, even for the same organism (e.g. a lactating female), the physiological and psychological state of the responder, and the relevance of the stress to the organism, can induce distinct neuroendocrine response and hence, result in different immune outcomes.

Stressors can also modulate oxytocin neurotransmission, which can attenuate ACTH and corticosterone responses (Neumann et al., 2000). Oxytocin modulates inflammation by decreasing the neuroendocrine and cytokine activation caused by bacterial endotoxin (Clodi et al., 2008). An oxytocin analog was even proposed as a potent modulator of inflammation, enhancing T cell activation in COVID-19 (Imami et al., 2020). Prolactin secretion by the pituitary, increases in response to psychosocial stress (Lennartsson and Jonsdottir, 2011), and correlates with immune system function by inducing dendritic cells to produce IL-6 and IL-23, which further alters T regulatory cell (Treg) phenotype, contributing to intestinal inflammation (Wu *et al*., 2014). Additional hormones such as thyroid-stimulating hormone (Jaeger et al., 2021), and somatostatin are all secreted by the pituitary, are affected by stress and are known to have immune modulating effects (Casnici et al., 2018; Jaeger et al., 2021).

Another system tightly linked to some forms of stress and especially relevant to immune regulation is the opioid system, which has well established effects on the immune system (Moyano and Aguirre, 2019; Karagiannis et al., 2020). Stress is known to elicit pain relief, a phenomenon referred to as stress-induced analgesia (Madden et al., 1977). In general, less severe stressors are thought to activate the endogenous opioid system and elicit the opioid-mediated form of stress-induced analgesia. Accordingly, this response can be blocked by naloxone, a non-selective opioid receptor antagonist (Bondar et al., 1978). In contrast, more severe stressors activate other pain inhibitory mechanisms that are independent of the endogenous opioid system (Lafrance et al., 2010). Effects of opiates on immune activity are well characterized (Moyano and Aguirre, 2019; Karagiannis *et al*., 2020). Thus, differential secretion of opiates is expected to induce distinct immune effects. Indeed, it has been shown that in rats subjected to one of two inescapable foot-shock stress paradigms, both of which induce analgesia, only one acts via activation of opioid mechanisms. The foot-shock paradigms are identical in shock intensity and total “shock on” time but differing in the temporal parameters of their application. Intermittent foot-shock causes an opioid-mediated analgesia; the continuous foot-shock cause an equally potent but nonopioid analgesia. Splenic natural killer cell activity is suppressed by the opioid, but not the nonopioid, form of stress. This suppression is also blocked by the opioid antagonist, naltrexone (Shavit et al., 1984). Similar suppression of natural killer activity is induced by high doses of morphine (Shavit *et al*., 1984). The duration of stress is also a factor in the activation of opioid or non-opioid mediated forms of stress-induced antinociception (Akil et al., 1985). Besides opioid receptors expressed on immune cells, the atypical scavenger receptor for chemokines ACKR3 or CXCR7, expressed on immune cells (Koenen et al., 2019) is a broad-spectrum scavenger for opioid peptides (Meyrath et al., 2020). Interestingly, immune cells have been shown to express opioid peptides such as β-endorphin, Met-enkephalin, and dynorphin A (Labuz et al., 2009). Thus, stressors induce different patterns of brain activity; hence, the inputs provided to the hypothalamic nuclei controlling hormone secretion, are distinct.

The SNS is a central neuronal mediator of the stress response. Sympathetic fibers reach all immune organs and the preganglionic cholinergic innervation to the adrenal gland (sympathetic–adrenal–medullary axis; SAM), inducing secretion of catecholamines adrenaline, and NA (Brede et al., 2003). These catecholamines are secreted directly into the bloodstream and are thereby delivered to the entire organism. In contrast to this systemic response, sympathetic fibers individually target almost every tissue in the body, including all primary and secondary lymphoid organs (Sloan et al., 2007; Maryanovich et al., 2018; Ding et al., 2019). The sympathetic fibers at one tissue can be activated independently of fibers at another site, locally releasing NA. This innervation of the lymphoid organs is dynamic, and it has been shown for example that chronic stress increases the sympathetic innervation to the lymph nodes (Sloan *et al*., 2007).

The local innervations also allow a corrective response to be initiated that is relevant to the specific stressors. Thus, for example, heat or cold stress, requires adaptation of the brown fat tissue to restore the temperature imbalance, while a scary sight requires adaptation of the cardiovascular system to allow effective escape, or priming of the immune system to promote tissue healing. Such specificity in regulating the different organs is enabled by sympathetic fibers that can be differentially activated in the cardiovascular system, the adipose tissue and the spleen, in response to different physical stressors (Iriki and Simon, 2012). Moreover, differences can be observed in the sympathetic activity of nerve fibers that innervate cells in the adrenal medulla that secrete adrenaline vs noradrenaline (Edwards et al., 1996). The differential activity can be regulated at multiple levels, either at the target site itself, the spinal cord or the brain, but all signals must be synchronized to execute an orchestrated response.

Another important aspect in our understanding of the local innervation of the SNS is that it is not limited to NA secretion. Peripheral nerve terminals co-secrete neuropeptides such as neuropeptide Y, vasoactive intestinal peptide (VIP), galanin, Substance P, neurotensin and others along with NA (Besedovsky and Rey, 2007; Chen et al., 2021). These neuropeptides, stored in varicosities along the axons (Shakiryanova et al., 2005; Zhao et al., 2011), are known to induce a range of immune effects, as many immune cells express the relevant receptors. However, the factors that govern this co-transmission are still unknown. In addition to brain-induced signals, it was suggested that local immune signals can locally regulate neuropeptide secretion (Talbot et al., 2015). The effects of neuropeptides on immune activity are increasingly gaining attention, especially in the context of gut-neuroimmune axis (Veiga-Fernandes and Mucida, 2016). Neuropeptides such as VIP or neuromedin U (NMU) in the gut have been shown to regulate the intestinal barrier by modulating innate lymphoid cells (ILC)3 (Seillet et al., 2020; Talbot et al., 2020) and ILC2 cells (Nussbaum et al., 2013; Cardoso et al., 2017). Another interesting effect has been shown to be mediated via eosinophils that can express CRH in the jejunum in response to psychological stress. Substance P induces CRH expression by these eosinophils, which in turn, activates mast cells to induce jejunum epithelial barrier dysfunction (Zheng et al., 2009). These interactions add another layer of complexity to neuroimmune communication in barrier tissues in general, although the studies examining these interactions in the context of stress are still limited.

The functional outcomes of the SNS cannot be viewed in isolation from the activity of its counterpart, the PaSNS. In the context of immune regulation, the PaSNS is known for its anti-inflammatory functions (Pavlov and Tracey, 2017) and it is one of the most extensively characterized pathways of brain-immune interactions. Thus, although the classical stress response is associated with an increase in SNS activity, regulation of the PaSNS can also impact the overall effect of the stress response on immunity. It has been shown for example that the skeleton, via the secretion of osteocalcin from osteoblasts, regulates the development of an acute stress response. Osteocalcin signals to post-synaptic parasympathetic neurons and inhibits their activity. This increases the relative impact of the sympathetic arm, which is left unopposed (Berger et al., 2019). In addition, acetylcholine (Ach), which can be produced by PaSNS neurons as well as immune cells, acts on these cells in an autocrine or paracrine manner, and it is mainly associated with the suppression of pro-inflammatory cytokines, such as tumor necrosis factor α (TNFα) (Pavlov and Tracey, 2017).

In summary, the endocrine profile and the specific activity of peripheral nerves is a reflection of the complexity of the brain’s overall response to the stress, which includes information regarding the physiological and psychological state of the organism, the specific pressure applied by the stressor, and the induced corrective response. Inputs from higher cortical areas, limbic structures and other hypothalamic nuclei converge to regulate the secretion of specific hormones, opiates and neuropeptides, and regulate the activity patterns of sympathetic and parasympathetic neurons. Signals conveyed by these mediators are ultimately integrated to affect the immune system. The form of the resulting immune activity depends on the end target, the immune cells and their environment (Fig 1C and Table 1).

## The target- cells of the immune system

**Figure 3:**
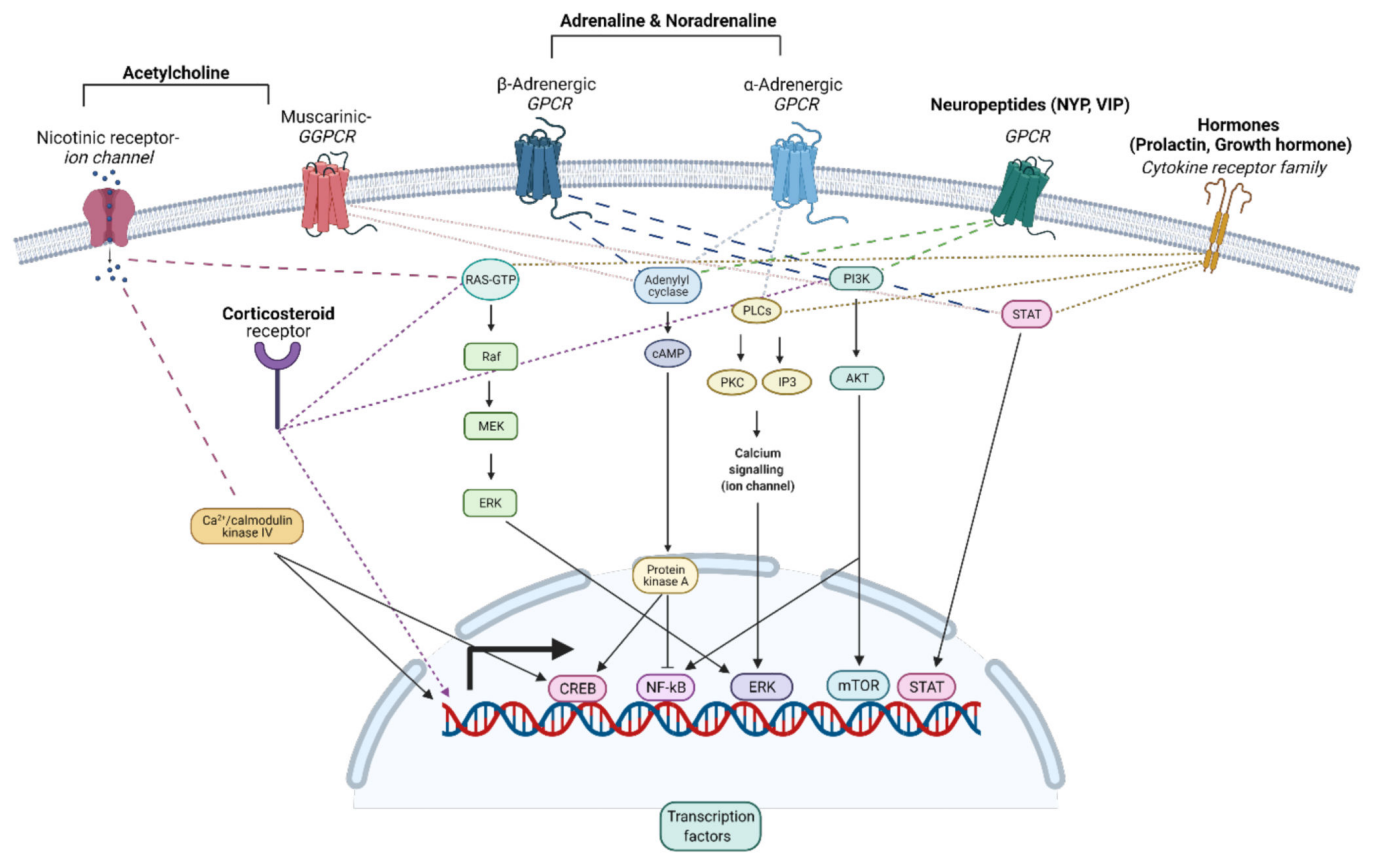
Intracellular immune cell-signaling by stress-induced mediators. Representative examples of receptors and their corresponding pathways that can be activated by stress-induced mediators. Immune cells express nicotinic and muscarinic receptors for acetylcholine, α- and β-adrenergic receptors for both adrenaline and noradrenaline, and members of the cytokine receptor family for hormones. Receptors for neuropeptides include mainly GPCR (e.g NPY, VIP), although exceptions exist, and the receptor for substance P, for example, is a member of the cytokine receptor family. Downstream signaling of these receptors regulates gene expression via transcription factors such as NF-κB, CREB, ERK, STAT, mTOR etc. Most of the receptors are expressed on the cell membrane, while corticosteroid receptors reside mostly within the cytosol. GPCR: G-protein-coupled receptor; cAMP: cyclic adenosine monophosphate; PLC: Phospholipase C; PKC: Protein Kinase C; IP3: Inositol trisphosphate; PI3K: Phosphoinositide 3-kinases; Akt: protein kinase B; NF-κB: nuclear factor kappa-light-chain-enhancer of activated B cell; CREB: cAMP response element-binding protein; ERK: extracellular signal-regulated kinases; STAT: signal transducer and activator of transcription; mTOR: mechanistic target of rapamycin.

The expression of receptors for neurotransmitters, neuropeptides and hormones on immune cells is well characterized (Glaser and Kiecolt-Glaser, 2005; Besedovsky and Rey, 2007). The cell type and its location determine the expression pattern of these receptors and their specific combinations. These receptors can counterbalance or synergize each other as they often modulate common downstream signaling pathways or different aspects of the same function (e.g cell motility) (Figure 3). Moreover, receptors can modulate each other’s expression and the sequence in which they are activated will eventually determine the functional outcome. For example, the opioid receptor, MOR, and the α2-adrenergic receptor communicate with each other through a cross-conformational switch that permits direct inhibition of one receptor by the other, with subsecond kinetics (Vilardaga et al., 2008). Morphine binding to the MOR triggers a conformational change in the NA-occupied adrenergic receptor (Vilardaga et al., 2008). Moreover, immune mediators, such as cytokines, modulate expression of receptors for neuromodulators. Thus, IL-2 induces up-regulation of β adrenergic receptor 2 (ADRB2) on CD8^+^ T cells (Wahle et al., 2001), increasing their sensitivity to the effects of NA stimulation. Given the immunosuppressive effects of NA, increasing the intrinsic expression of ADRB2 may provide the cells with an additional layer of modulation that can prevent over activation and limit collateral damage during effector responses. Currently, no comprehensive studies have directly characterized these relationships, or compared the overall expression patterns across different immune populations, sites, and the dynamics of their expression following different stimuli.

The emergence of public databases that integrate RNA sequencing data from multiple laboratories allows some of these questions to be addressed with greater precision and to estimate the expression of receptors for neuromodulators on different immune cells. Overall, there are multiple receptors for NA, α-adrenergic and β2-adrenergic receptors, all of which are G-protein-coupled receptors (GPCR). For Ach, there are ionotropic, nicotinic and G-protein-coupled muscarinic receptors. Most receptors for both NA and Ach are expressed on immune cells, and the ADRB2 is the most abundant receptor (Besedovsky and Rey, 2007; Chen *et al*., 2021). The amounts of specific receptors vary between cell subsets, and receptors are expressed on both innate and adaptive immune cells. Adrenergic receptors appear to have highest expression on innate immune cells, while the nicotinic Ach receptors are more diverse in their expression patterns and are also abundant on CD4^+^ and B cells (Chen *et al*., 2021). Notably, with the emergence of single cell sequencing data, it has become clear that although cells can be broadly clustered based on their expression patterns, individual cells may express unique combinations of receptors. Thus, gene signatures from single-cell sequencing data (Cell-ID) (Cortal et al., 2021) can provide a new perspective on the specific combinations of receptors expressed by individual cells. Moreover, spatial transcriptomics will expand the ability to characterize differences between immune cells located in different niches, and their proximity to nerve fibers.

Functionally, neuronal mediators have been shown to induce a broad spectrum of immune responses, including cell activation, differentiation, motility and apoptosis (Besedovsky and Rey, 2007(Chen *et al*., 2021). Here, we concentrate on adrenergic receptor ligands that are the most abundantly expressed on immune cells, as previously mentioned. In general, ADRB2 signaling can suppress inflammatory cytokine secretion from dendritic cells T cells (Estrada et al., 2016) and innate lymphoid cells (Agac et al., 2018; Moriyama et al., 2018). Several mechanisms have been associated with the immunosuppressive functions of beta adrenergic signaling, including inhibition of NF-kB activation (Pavlov and Tracey, 2017), induction of immune suppressive cytokines, mainly IL-10 (Agac *et al*., 2018), and promotion of Treg cell devolvement by inducing FOXP3 expression in CD4^+^ T cells (Guereschi et al., 2013). In addition to its suppressive function, NA also has proinflammatory effects on some cell subsets. For example, ADRB2 signaling enhances natural killer (NK) cell expansion and effector function *in vivo* in response to virus infection (Diaz-Salazar et al., 2020). Moreover, NA is known to affect immune cell trafficking (Xiu et al., 2013; Moriyama *et al*., 2018), mobilizing immune cells to the blood in response to stress (McKim et al., 2018).

The activity of the different receptor subtypes can drive distinct cellular responses. For example, activation of β2-adrenergic receptors drives anti-inflammatory (M2) macrophagedevelopment (Grailer et al., 2014), while α-adrenergic receptor activation increases TNFα production by macrophages (Huang et al., 2012). β adrenergic receptors can be up-regulated on NK cells in early inflammation, inducing NK cell expansion and memory (Diaz-Salazar *et al*., 2020). Inflammation can also affect the glucocorticoid receptor expression profile, suggesting that stressor timing (considering the state of the organism) can induce varied cellular responses. Similarly, duration and intensity of the signal can produce different responses. Extended treatment of bone marrow-derived myeloid cells with NA can inhibit their maturation, and high NA concentrations inhibit expression of MHCII and the CCR2 chemokine receptor, and enhance TNFα expression on these cells (Xiu *et al*., 2013).

In terms of endocrine mediators, the HPA axis mediates the secretion of glucocorticoids and mineralocorticoids from the adrenal gland cortex to the blood. Immune cells are affected by both hormones through their binding to intracellular steroid receptors, and signal transduction, which leads to a change in gene expression via transcription factor binding to DNA (Figure 3). Glucocorticoids are the most tightly linked to immune system function, and the glucocorticoid receptor (Cain and Cidlowski, 2017) regulates gene transcription with temporal dynamic effects on innate and adaptive immunity (Cain and Cidlowski, 2017). Glucocorticoids enhance the transcription of genes encoding toll-like-receptor (TLR) inhibitors (Su et al., 2017). However, upon prolonged exposure, glucocorticoids inhibit expression of integrins and trafficking molecules (E-selectin, Icam, Ccl2, Cx3cl10) thereby reducing immune cell recruitment (Kressel et al., 2020). A recent paper has shown that FOXp3^+^Treg cells are key mediators of glucocorticoid-based treatment effects in a multiple sclerosis model (EAE), acting through miR-342-dependent metabolic control in Treg cells (Kim et al., 2020). In contrast to these well characterized anti-inflammatory properties of glucocorticoids, exposure at low doses and/or before antigen challenge can enhance the inflammatory response (Duque Ede and Munhoz, 2016; Macfarlane et al., 2020). For example, glucocorticoids drive diurnal oscillations in T-cell distribution and responses by inducing IL-7 receptor and CXCR4, and therefore, enhance adaptive immunity (Shimba et al., 2018).

The targets of the stress response are not only immune cells. To alter immune activity in response to stress, the brain can either directly affect the immune cells themselves or regulate the environment in which these cells function (Fig 2). For example, sympathetic and endocrine signals modulate the blood vessels through which immune cells travel (Devi et al., 2021). NA can directly induce MAdCAM-1 expression on the endothelial cells, reducing the extravasation of immune cells from the blood to tissues (Schiller et al., 2021a) or induce vasoconstriction and decreased local blood flow to attenuate cell motility (Devi *et al*., 2021). Glucocorticoids have also been shown to modulate the expression of adhesion molecules on endothelial cells such as ICAM1, CCl2, and CXCL10 (Kertser et al., 2019), altering immune cell interactions in the choroid plexus. In other tissues, such as the bone marrow, mesenchymal cells in the hematopoietic niches respond to NA, via β3 adrenergic receptors, and downregulate CXCL12, inducing mobilization of hematopoietic progenitors from the bone marrow to the blood (Heidt et al., 2014). Brown fat tissue adipocytes initiate a chain of events mediated by adrenergic signaling and IL-6 release which metabolically fuels adaptive “fight or flight” responses (Qing et al., 2020). In the gut, NA and corticosterone have been shown to affect microbiota composition, which in turn, can affect immunity (Karl *et al*., 2017; Gao *et al*., 2018; Rengarajan et al., 2020). Additionally, microbiota can modulate sympathetic activity in the gut (Muller et al., 2020). Thus, the brain can regulate stress-related immune activity through direct effects, or by altering the environment in which immune cells develop and function. The expression of the various receptors for neuromodulators is very dynamic and vary with the activation and developmental state of the cells. Thus, the functional outcome of stress depends on multiple factors, including cell type, receptor subtype, activation/inflammatory state, exposure duration, signal concentration, and combined activities of tissue and ligand pairs (Fig 1C). This functional complexity of the neuromodulatory system on the immune response is far from being fully understood, and the combinatorial intricacies embedded in diversity of their potential outcomes poses a major challenge to this field.

## Concluding remarks

Understanding stress is essential for our understanding of physiology. Here, we propose that the diversity of immune responses to stress reflects a range of physiological demands imposed by the stressor. Therefore, stressors cannot be treated as a uniform entity. We reviewed here a series of factors that can contribute to the categorization of the stressors. However, even distinctions that seem obvious, such as the difference between psychological and physiological stress, are not trivial, as most experiences are comprised of some combination of both. For example, the foot shock paradigm, is used by some researchers as a model of physical stress (Li *et al*., 2019), but is considered psychological stress by others (Kertser *et al*., 2019). The distinction between acute and chronic stress, as well as their definitions, are also not trivial, as chronic stress is comprised of both prolonged exposure to a stressor, and the psychological anticipation and prediction of the stressor outcome. Thus, there is a fundamental difference between chronic repeated stress, in which the same stressor is applied repeatedly, and chronic unpredictable stress, in which the stressor and the time of its application change between experiences or the combination of physical and psychological stress (Du Preez *et al*., 2020).

In this review we attempted to describe the specific components that enable generating a diverse range of immune effects in response to different stressors. Nevertheless, it should be noted that physiological reactions (e.g immune response, metabolism, cardiovascular changes) cannot be viewed in isolation, and should be synchronized with behavioral outputs. Moreover, immune cell activity is not limited to coping with exposure to pathogens or potential tissue damage during stress. Immune cells are required to enable the effective activity of other physiological systems required to cope with the stressor. For example, in the heart, cardiac macrophages are essential for survival during cardiac stress, maintaining electrical conduction through the regulation of cardiac gap junction formation (Sugita et al., 2021). Hypertension, a common response to stress, has a strong immune component, and hypertension cannot be induced in immune-deficient mice (e.g RAG and SCID) (Carnevale et al., 2014). Increased respiration is induced in some stress responses and is supported by the activity of alveolar macrophages (Broug-Holub et al., 1998). Metabolism, one of the requirements of adaptation to the stress imposed on the organism, is also dependent on immune activity. For example, macrophages in adipose tissue influence the metabolic balance of an organism by modulating its glucose tolerance, lipid uptake and thermogenesis (Wolf et al., 2017; Jaitin et al., 2019), as well as regulating the neuronal mediators. Specifically, sympathetic neuron-associated macrophages in the adipose tissue have been shown to mediate clearance of NA, thereby playing an important role in conversion of white fat to brown, and thermogenesis (Pirzgalska et al., 2017). Thus, to generate an effective and orchestrated response of the organism to stress, the immune response has to be synchronized to support these diverse physiological functions. Moreover, the brain can anticipate the need for these physiological changes, and upon detection of the stressor, prime the relevant immune activity (Godoy *et al*., 2018).

We focused here on how nervous system activity during stress affects immunity. However, this is an artificial distinction as stress affects different physiological systems and should be studied from a multisystem perspective. For example, hyperarousal and insomnia induced by restraint stress is mediated via activation of hypocretin neurons in the lateral hypothalamus and the CRH neurons innervated by them. Optogenetic stimulation of the same CRH neurons induces changes in immune cell distribution and their functional responses (e.g., reduction in circulating CD4^+^ T cells and an increase in IkB expression) (Li et al., 2020). In fact, some of the long term and complex effects of stress on physiology can be elucidated only from a systemic perspective. Examining the complex multi-directional interactions between the brain and the periphery can explain some of the adverse effects that stressors, such as psychological stress, have on various human diseases, including those of the cardiovascular system. For example, in a vascular disease model of vaso-occlusion in sickle cell disease, it has been shown that stress promotes vaso-occlusion by eliciting a glucocorticoid hormonal response that augments gut permeability, leading to microbiota-dependent IL-17A secretion from T helper 17 cells of the lamina propria, followed by the expansion of the circulating pool of aged neutrophils that trigger vaso-occlusions (Xu et al., 2020).

Moreover, peripheral changes, specifically changes in immune activity, affect the nature of the sensory information delivered to the CNS, influencing the perception and evaluation of the stressor and its potential implications within the brain. For example, immune activity alters the sensitivity of sensory fibers and pain signals (Pinho-Ribeiro et al., 2018). Thus, these are bi-directional relationship as neuroendocrine stimuli influence immune function while immune mediators can mediate sensory information and hence, neuroendocrine signals.

Changes in neuronal activity affect the brain’s own immune compartment, composed of resident and infiltrating immune cells. Neurotransmitters, neuropeptides and hormones have been shown to affect microglia, which in turn, affect the brain’s stress response. Microglia are responsive to hormones and neurotransmitters induced during stress, such as corticosterone and NA (Wohleb et al., 2013; Horchar and Wohleb, 2019; Bollinger et al., 2020).

The selected examples provided in this review do not represent the full complexity of the stress response; however, they emphasize the breadth of systemic analysis required to approach it. Studying such systemic reactions carries the challenges of integrative physiology, which requires a deep understanding of the processes at each system, as well as the interactions between them. Indeed, current knowledge gaps are evident at each level. At the neuroscience level, the representation of different stressors is not fully characterized, and our understanding of the control over the hypothalamic and brainstem areas that regulate the outflow from the brain is still limited. In terms of mediators, it is known that peripheral nerves can co-secrete neuropeptides; we do not know, however, how this co-transmission is regulated. We also do not fully understand the peripheral interactions between the sympathetic and parasympathetic nervous systems. In terms of the interactions between the mediators and the immune system, many aspects of these relationships are not yet understood, especially in the case of psychological stress. Thus, the factors regulating the expression of specific receptors on immune cells remain unknown, the dose dependence of the activity of the different mediators has not been characterized in detail, and how different combinations of mediators affect function is yet to be determined.

Filling these gaps will contribute to uncovering the mechanics of the stress response induced by the brain. However, to overcome, at least in part, the contradictions and confusion in the field, we must transition from a uniform approach to stress, to defining it based on its physiological relevance. For each stress paradigm, we should define the strains it puts on the organism, the neuro-matrix it induces, and the peripheral output associated with the specific stressor and the elicited immune respnse. In the field overall, we will need to define specific parameters that will be used to evaluate each stressor and stress paradigm, define a relevant terminology that will allow us to distinguish between different forms of stress and then evaluate the functional effects in terms of their immunological impact. Since the effects of stress on the immune system take place at multiple levels (systemic/local; central/peripheral), they require reciprocal interactions between different physiological systems, many of which we are just beginning to uncover. While these knowledge gaps pose a major challenge for stress research as a field, it is also clear that understanding stress is an essential route in our quest to improve human health.

## Figures and Tables

**Table 1:** Potential diversity of induced stress responses and their immunological outcome. Summary of studies focused on psychological, physical and combined forms of stress when presented acutely or chronically. Chronic physical stress includes both physical and psychological components. We provide examples of how different stressors can affect each component of the stress response (initiator/brain, mediator/neuronal and endocrine, recipient/immune cells) as well as representative effects on the immune system. The data presented were compiled from a collection of independent papers, with each referring to a specific type of stress and an associated effect either on the brain, the mediators, theimmune system, or their combination. The lack of more comprehensive studies that include all aspects of the stress response emphasizes the need for broader analyses in order to gain a coherent mechanistic understanding of the immunological effects of stress. NK- natural killer cells, DCs- dendritic cells, BMSCs- bone marrow stem cells

Origin	Initiator	Mediators	Targets	Effects
**Psychological**	Hippocampus (Goldfarb et.al., 2020)	Corticosterone (Debahr & mcEwen 1997, Abe et.al., 2017)	Lymphocytes, BMSCs, DCs neutrophils, monocytes and macrophages	Enhanced cell-mediated immunity (Debahr & mcEwen 1997) Anti-inflammatory reflex (Abe et.al.,2017)
	Insular cortex (Limbachia et.al., 2021)			
	Hippocampus (Kim and Cho, 2020)	Sympathetic nervous system (Solan et.al., 2007)	Lymphocytes, BMSCs, monocytes and macrophages	Regulatory T cell and DC dysfunction (Wu et.al., 2014) Enhanced monocyte recruitment to the brain (Wohleb et.al.,2013)
	Amygdala (Kim and Cho, 2020)	Prolactin (Wu et.al., 2014, Lennartsson and Jonsdottir et.al., 2011)	Lymphocytes, granulocytes, monocytes and macrophages	
	Cingulate cortex (Jeon et al., 2010)			
**Psychological and physical**	Prefrontal cortex (Amat et al., 2005)	Growth hormone (Jezova et.al., 2006)	Lymphocytes, monocytes and macrophages	Peripheral blood mononuclear cell and BMSC apoptosis, in a corticosterone dosedependent manner (Sarjan & Yajurvedi, 2018)
	Nucleus accumbens (Hodes et.al., 2015)	Corticosterone, ACTH (Sarjan & Yajurvedi, 2018)	Lymphocytes, BMSCs, DCs neutrophils, monocytes and macrophages	
**Physical**	Hypothalamus: Paraventricular nucleus (Babb et.al., 2013) Arcuate nucleus (Lookingland et.al, 1991)	Substance P (Pavlovic et.al.,2011)	T and B cells, eosinophils, mast cells, monocytes and macrophages	Suppressed cell-mediated immunity (Debahr & mcEwen 1997) Increased DC maturation and frequency (Pavlovic et.al.,2011) Increased neutrophil and lymphocyte mobilization into the blood, followed by decreased tissue trafficking of all cell types (Dhabhar et.al., 2012)
		Corticosterone and ACTH (Zelena et.al., 2009, Babb et.al., 2013)	Lymphocytes, BMSCs, DCs neutrophils, monocytes and macrophages	
		α-melanocyte-stimulating hormone (Lookingland et.al, 1991)	Lymphocytes, basophils, neutrophils, monocytes and macrophages	
Acute	Chronic			

## eTOC

Stress has diverse and often contradictory effects on immune activity. Rolls et al discuss potential mechanisms whereby different stressors, which induce distinct patterns of brain activity, modulate the neuroimmune axis.
